# MEX3A promotes nasopharyngeal carcinoma progression via the miR-3163/SCIN axis by regulating NF-κB signaling pathway

**DOI:** 10.1038/s41419-022-04871-0

**Published:** 2022-04-30

**Authors:** Xin-xin Xiang, Yong-liang Liu, Yi-fan Kang, Xiang Lu, Kai Xu

**Affiliations:** 1 Center of Translational Medicine, Zibo Central Hospital, Shandong University, Zibo, 255036 China; 2 Department of Otolaryngology, Zibo Central Hospital, Shandong University, Zibo, 255036 China; 3 Department of Otorhinolaryngology Head and Neck Surgery, Tongji Hospital, Tongji Medical College, Huazhong University of Science and Technology, Wuhan, 430030 China

**Keywords:** Head and neck cancer, Mechanisms of disease, Head and neck cancer

## Abstract

Mex-3 RNA Binding Family Member A (MEX3A) is an RNA-binding protein that plays complex and diverse roles in the development of various malignancies. However, its role and mechanism in nasopharyngeal carcinoma (NPC) remain undefined and were therefore evaluated in this study. By analyzing Gene Expression Omnibus data and using tissue microarrays, we found that MEX3A is significantly upregulated in NPC and negatively associated with prognosis. Notably, MEX3A depletion led to decreased cell proliferation, invasion, and migration, but increased apoptosis in NPC cells in vitro, while inhibiting tumor growth in vivo. Using whole-transcript expression arrays and bioinformatic analysis, we identified scinderin (SCIN) and miR-3163 as potential downstream targets of MEX3A in NPC. The regulatory mechanisms of MEX3A, SCIN and miR-3163 were further investigated using rescue experiments. Importantly, SCIN depletion and miR-3163 inhibition reversed and rescued the oncogenic effects of MEX3A, respectively. Moreover, NF-κB signaling inhibition reversed the oncogenic effects of both SCIN and MEX3A. In summary, our results demonstrate that MEX3A may promote NPC development and progression via the miR-3163/SCIN axis by regulating NF-κB signaling, thus providing a potential target for NPC treatment.

## Introduction

Nasopharyngeal carcinoma (NPC), a cancer arising from the nasopharyngeal epithelium, has remarkable ethnic and geographic distribution and is highly prevalent in southeast Asia and northern Africa [1, 2]. The incidence of NPC is 3 per 100 000 in China and 0.7 per 100 000 globally [2]. Despite similar anatomical or tissue lineage origins, NPC is distinctly different from other head and neck cancers [1]. Because of the obscure initial symptoms and high invasive and metastatic potential of NPC, more than 70% of patients with NPC are diagnosed only at an advanced stage and often with unsatisfactory prognoses [3, 4]. Although NPC is sensitive to radiotherapy, 10–20% of patients with NPC experience distant metastases or local or regional recurrence after radiotherapy, contributing to NPC treatment failure and cancer-related mortality [4, 5]. These facts clearly indicate that NPC treatment should focus not only on pursuing good loco-regional control, but also on preventing distant relapse and prolonging remission in patients with metastatic disease. To this end, innovative strategies based on new tumor markers and therapeutic targets are urgently needed.

However, NPC is inadequately understood at the molecular level [6]. Crucial genomic changes, including various loss-of-function mutations in NF-κB negative regulators [7, 8] and recurrent genetic lesions, including loss of the *CDKN2A*/*CDKN2B* locus, *TP53* mutation, *CCND1* amplification, chromatin modification, and mutation in the PI3K signaling pathway [1, 6], promote NPC development and progression. Nevertheless, genomic abnormalities in NPC remain largely undefined, and no effective therapies have been established [9]. Therefore, it is important to characterize genomic variations in NPC and identify key genes involved in metastasis and recurrence to guide the development of new therapeutics [1, 6].

Aberrant expression of RNA-binding proteins is commonly seen during the progression of human cancers [10]. Dysregulated RNA-binding proteins often promote oncogenic isoforms that affect cell proliferation and invasion [10, 11]. Mex-3 RNA Binding Family Member A (MEX3A) is an RNA-binding protein that regulates gene expression at the post-transcriptional level [12]. MEX3A is involved in embryonic development, epithelial homeostasis, and tumorigenesis [12–14]. Further, MEX3A plays complex and diverse roles in the development of various malignancies, including colorectal cancer [13], lung cancer [15], liver cancer [16], cervical cancer [17], and glioma [18]. The specific mechanisms of MEX3A in tumorigenesis seem to be tissue- and context-dependent [15, 17, 18]. However, whether MEX3A plays a role in NPC remains undetermined.

In this study, we aimed to unravel the role and potential mechanism of MEX3A in NPC through bioinformatics analyses, tissue microarrays (TMAs), and loss-of-function studies in vitro and in vivo. Whole-transcript expression array and ingenuity pathway analysis (IPA) were employed to establish the underlying molecular mechanism of MEX3A in NPC. Further, IPA was used to examine potential downstream targets of MEX3A, including scinderin (SCIN), an actin-severing protein involved in the development and progression of several malignancies [19–22]. Our results highlight an important role of MEX3A in the oncogenesis of NPC, suggesting a novel therapeutic target and prognostic marker of NPC.

## Materials and methods

### Clinical samples and tissue microarray (TMA)

Two RNA-seq datasets of NPC and non-NPC tissues were downloaded from Gene Expression Omnibus (GEO) database (GSE53819 and GSE12452). Formalin-fixed NPC TMAs (HNasN132Su01) and the para-carcinoma normal tissue chip were purchased from Shanghai Outdo Biotech Company Co., Ltd. (Shanghai, China). 132 NPC tissue samples were collected between January 2010 and October 2011, and included in TMAs (HNasN132Su01). Patient characteristics and survival data in TMAs are listed in Table S2 (*n* = 105) and Table S5 (*n* = 128) up to the number of sections detached during immunohistochemistry, and written informed consent were obtained from all participants. The study was approved by the Human Research Ethics Committee of Tongji Hospital, Tongji Medical College, Huazhong University of Science and Technology.

### Immunohistochemistry and antibodies

TMAs and tissue sections were dewaxed, dehydrated, endogenous peroxidase blocked, and antigens retrieved according to standard procedures. The sections were incubated with diluted primary antibody at 4 °C overnight and continuously with the indicated secondary antibody at 37 °C for 1 h. Finally, the tissue sections were stained with diaminobenzidine and examined using CaseViewer_2.0_RTM software v2.0.2.61392 (3DHISTECH Ltd., Budapest, Hungary). Images of representative fields were acquired using the Aperio ImageScope software v11.2.0.780 (Leica Biosystems, Wetzlar, Germany). Immunohistochemical staining was evaluated based on the H-score as previously described [23], and conducted by two independent experienced pathologists. High or low grading was based on the median H-score of all tissue samples.

All antibodies used in this study are listed in Table S7.

### Cell lines and culture

The 293 T and human NPC cell lines (CNE-2Z, C666-1, HONE-1, and 5–8 F) were purchased from the American Type Culture Collection (ATCC, Manassas, VA, USA). All cells were cultured in RPMI-1640 medium (Corning Inc., Corning, NY, USA) containing 10% FBS (Gibco, Rockville, MD, USA) at 37 °C in a humidified 5% CO_2_ atmosphere. C666-1 and HONE-1 cells at approximately 80% confluence were cocultured with lentiviruses carrying target sequences and a green fluorescent protein sequence for 72 h. Lentivirus infection efficacy was evaluated using a fluorescence microscope (Olympus, Tokyo, Japan). NF-κB inhibition was conducted by incubating cells with 20 nM QNZ (EVP4593) (Selleck, Houston, TX, USA) for 24 h, DMSO solution was used for negative control.

### Plasmids and transfection

To generate lentiviral short hairpin (sh)RNA constructs targeting human SCIN and MEX3A, the indicated target sequences (Table S8) were cloned into the pLKO.1-puro lentiviral vector (Addgene, Watertown, MA, USA). MEX3A and SCIN overexpression constructs were generated by cloning MEX3A and SCIN sequences into pCDH-MSCV lentivirus (Shanghai Biosciences, Shanghai, China). The packing of indicated shRNA and pCDH-MSCV lentiviral particles was conducted as previously described [24].

### RNA extraction and RT-PCR analysis

RNA extraction and cDNA synthesis were carried out using TRIzol reagent (Invitrogen, Carlsbad, CA, USA) and the PrimeScript RT-PCR kit (Takara Bio, Shiga, Japan). qPCRs were performed using the primers listed in Table S9 and the SYBR Green Master Mix Kit (Vazyme, Nanjing, China) on a VII7 Real-Time PCR System (ABI, Waltham, MA, USA). *GAPDH* was used as an internal control. Gene expression levels were calculated using the 2^−ΔΔCt^ method.

### Western blotting and co-immunoprecipitation (co-IP) assay

Cells were lysed in ice-cold RIPA buffer (Millipore, Temecula, CA, USA) containing protease inhibitors (Roche Diagnostics, Basel, Switzerland). Protein concentrations were measured using a BCA Protein Assay Kit (HyClone-Pierce, Logan, UT, USA). The proteins were resolved by 10% SDS-PAGE (Invitrogen) and transferred to PVDF membranes. The membranes were immunoblotted and visualized as previously described [24]. All uncropped images are listed in Figure S7.

For the endogenous co-IP assays, protein lysates (1 mg) were incubated with the appropriate antibody (1–2 µg) at 4 °C overnight and then with 20 μL of Protein A Sepharose beads (Millipore) at 4 °C for 2 h. The total cell lysates and immunocomplexes were resolved and immunoblotted as previously described [24].

All indicated antibodies used are listed in Table S7.

### MTT assay

The MTT assay was performed as previously reported [23]. Briefly, shCtrl and shMEX3A stable C666-1 and HONE-1 cells were seeded in 96-well plates at 2000 cells/well. After incubation at 37 °C for 24, 48, 72, 96, and 120 h, the cells were incubated with 20 μL MTT solution (5 mg/mL; Genview, El Monte, CA, USA) for 4 h. After termination of the reaction, the optical density at 490 nm was measured using a Tecan Infinite microplate reader (Tecan, Zürich, Switzerland).

### Flow-cytometric analysis

Cells were seeded in six-well plates in triplicate and cultured for 5 days. Floating cells were collected, trypsinized, and washed with ice cold D-Hanks solution. After centrifugation, the cells were resuspended in binding buffer and stained with 10 μL Annexin V-APC (eBioscience, San Diego, CA, USA). Cell apoptosis was analyzed using a FACSCalibur flow cytometer (BD Biosciences, San Jose, CA, USA).

### Wound-healing assay

Cells were seeded in a 96-well plate at 3 × 10^4^ cells/well. When the cells reached >90% confluence, scratches crossing the cell monolayer were made using a 96 Pin Replicator (VP Scientific, San Diego, CA, USA). After gently rinsing the cells with RPMI-1640 medium 2–3 times, 0.5% FBS RPMI-1640 was added, and the cells were cultured for 24 h or 48 h. The scratch lines were observed under a fluorescence microscope and cell migration rates were calculated.

### Transwell assay

The Transwell assay was conducted using the Corning Transwell Kit (Corning Inc.) according to the manufacturer’s instructions. Briefly, cells were collected, trypsinized, counted, and incubated in the upper chamber with or without Matrigel in 100 μL FBS-free medium (5 × 10^4^ cells/well). Medium containing 30% FBS (600 μL) was added to the lower chamber. After incubation for 24 h (migration assay) or 48 h (invasion assay), the cells that migrated through the upper membrane were fixed, stained with 400 μL Giemsa stain, viewed under an inverted microscope, and counted.

### Celigo cell counting and Cell Counting Kit-8 (CCK-8) assays

Cells in the logarithmic growth phase were washed, trypsinized, seeded into 96-well plates (2 × 10^3^ cells/well), and cultured in RPMI-1640 containing 10% FBS at 37 °C in a 5% CO_2_ atmosphere. For the Celigo-based cell counting assay, as described previously [25], the cells were counted using a Celigo image cytometer (Nexcelom Bioscience, Lawrence, MA, USA) on days 1, 2, 3, 4, and 5. For the CCK-8 assay, after 24 h or 48 h, the cells were incubated with 10 μL CCK-8 solution (Sigma-Aldrich, St. Louis, MO, USA) for 2 h. The absorbance of the solution was measured at 450 nm using a Cellometer Mini Cell Counter (Nexcelom Bioscience).

### Colony formation assay

shCtrl and shMEX3A stable C666-1 and HONE-1 cells in the logarithmic growth phase were seeded into six-well plates (1 × 10^3^ cells/well) in triplicate and cultured for 8 days. Subsequently, the cell colonies were fixed with 4% paraformaldehyde, stained with Giemsa stain, photographed using a digital camera, and counted.

### Dual-luciferase reporter assay

The wild-type (WT) or mutant (Mut) sequences of MEX3A 3’-UTR or SCIN 3’-UTR were synthesized and separately inserted into psiCHECK™-2 vectors (Promega, Madison, WI, USA). The miR-3163 mimics, miR-3163 inhibitor, and negative control were constructed (GenePharma, Shanghai, China) and co-transfected with reporter plasmids into C666-1 cells using Lipofectamine 3000 (Invitrogen). The cells were harvested after 48 h and the luciferase activity of the treated cells was investigated using the dual-luciferase assay system (Promega), according to the manufacturer’s protocol.

### Expression analysis with microarrays

Gene expression profiling of shCtrl and shMEX3A stable C666-1 cells was conducted using the Human Clariom^TM^ S Array (Thermo Fisher Scientific, Waltham, MA, USA). Briefly, the quality and integrity of the extracted RNA from cells was evaluated using a Nanodrop 2000 spectrophotometer (Thermo Fisher Scientific). Preparation and hybridization of the samples were conducted using the GeneChip Whole Transcript PLUS Reagent Kit (Thermo Fisher Scientific) according to the manufacturer’s instructions. The data were scanned using the GeneChip™ Scanner 3000 (Affymetrix, Santa Clara, CA, USA) and analyzed using Transcriptome Analysis Console software v. 4.0.2 (Affymetrix), applying Welch’s *t*-test with the Benjamini-Hochberg correction. Genes with |Fold change | ≥ 1.3 and a false discovery rate < 0.05 were considered differentially expressed genes (DEGs). IPA (Qiagen, Hilden, Germany) was used to analyze DEG functions and interactions, and a | Z-score | > 2 was considered significant.

### Animal xenograft study

Female BALB/c nude mice (4 weeks old) were purchased from Beijing Charles River Experimental Animals (Beijing, China), and single blind randomly divided into shMEX3A (*n* = 10) and shCtrl (*n* = 10) groups for the xenograft experiments. shMEX3A or shCtrl stable C666-1 cells (4 × 10^6^ cells in 200 µL PBS) were subcutaneously injected into the dorsal flanks of mice for tumor development. Tumor growth was monitored by recording the longest dimension (L) and dimension perpendicular to L (W) once weekly and calculating the tumor volume (V = π/6 × L × W^2^). In vivo fluorescence images were captured after intraperitoneal injection of D-luciferin (15 mg/mL) using the IVIS Spectrum In Vivo Imaging System (Perkin Elmer, Waltham, MA, USA). All mice were sacrificed at 51 days post-injection. Mice tumor tissues were dissected, weighed, processed, stained with hematoxylin and eosin (H&E), and subjected to Ki-67 staining. The animal experimental design was reviewed and approved by the Institutional Animal Care and Treatment Committee of Huazhong University of Science and Technology.

### Statistical analysis

The sample size in each experiment was chosen to ensure adequate power to detect a pre-specific effect. The results of at least three independent experiments were qualified by blinded observers and expressed as the mean ± standard deviation (SD). Means of two groups were compared using the two-tailed Student’s *t*-test, while those of multiple groups were compared by one-way ANOVA. Differences in MEX3A expression between nasopharyngeal tissues and adjacent normal tissues were analyzed using rank sum tests. Correlations between MEX3A expression and patient characteristics were analyzed using Mann–Whitney *U* analysis and Spearman rank correlation analysis. Correlations between MEX3A and SCIN expression in NPC TMAs were analyzed using Spearman rank correlation analysis. *P* < 0.05 was considered statistically significant. All analyses were conducted using IBM SPSS Statistics software v 26.0 (IBM, Corp., Armonk, NY, USA) and GraphPad Prism software v8.0.2 (GraphPad Software, La Jolla, CA, USA).

## Results

### MEX3A is highly expressed in NPC and associated with the clinical stage

**Fig. 1:**
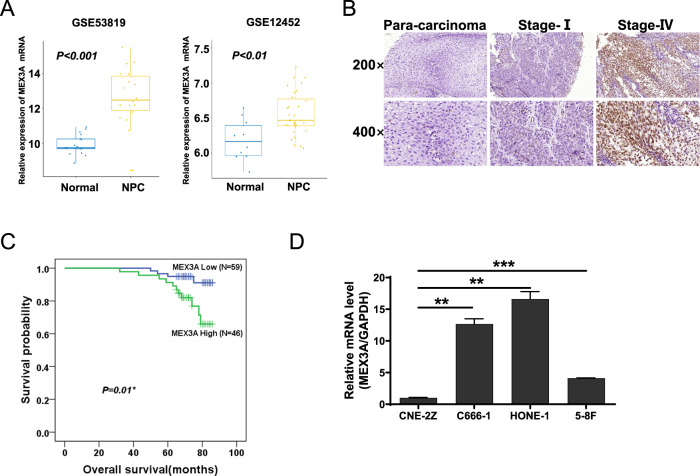
MEX3A expression is high in NPC, especially in the advanced stage. **A** MEX3A mRNA levels were obtained from GEO datasets (GSE53819, containing 18 normal nasopharyngeal tissues and 18 NPC tissues; GSE12452, containing 10 normal nasopharyngeal tissues and 31 NPC tissues). MEX3A mRNA levels were compared between the two groups using the Wilcoxon signed-rank test. **B** Compared with normal nasopharyngeal tissues, MEX3A levels were higher in NPC tissues, especially in the advanced stage group. **C** Kaplan–Meier curves of overall survival of NPC patients with high or low MEX3A expression (*n* = 105). **D** MEX3A mRNA levels in various NPC cell lines were detected by RT-qPCR. **P < 0.01, ***P < 0.001.

Analyzing the NPC GEO datasets revealed that MEX3A mRNA levels in NPC tissues were significantly higher than those in adjacent non-cancerous tissues (*P* < 0.01 or 0.001, Fig. 1A). We confirmed the association between high MEX3A expression and NPC using tissue microarrays (TMAs) (Fig. 1B, Table S1). Two patient characteristics included in TMA, sex and clinical stage, were significantly correlated with MEX3A expression (*P* < 0.05, Tables S2 and S3). The Kaplan–Meier survival curves indicated that increased MEX3A expression was significantly associated with worse prognosis (*P* < 0.05, Fig. 1C). Next, we determined MEX3A expression in a panel of NPC cell lines. As shown in Fig. 1D, C666-1 and HONE-1 NPC cells had higher MEX3A mRNA levels than CNE-2Z and 5–8 F cells. Therefore, these two cell lines were selected for subsequent experiments.

### MEX3A knockdown leads to increased cell proliferation and migration as well as decreased apoptosis in NPC cell lines

**Fig. 2:**
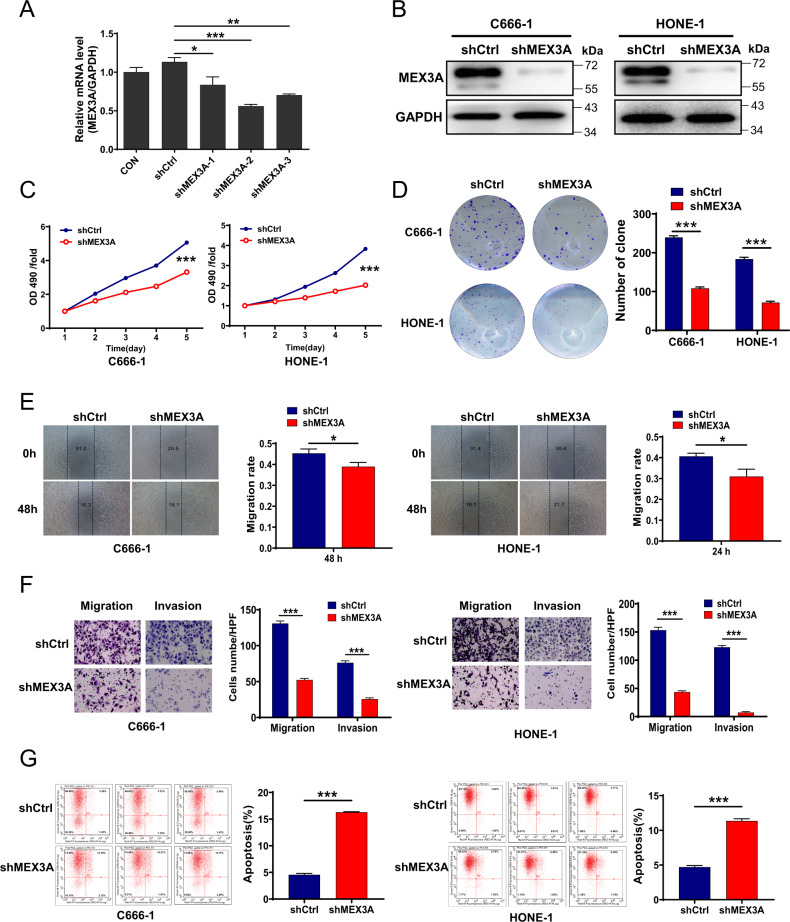
MEX3A knockdown leads to decreased proliferation, migration, and invasion as well as increased apoptosis in NPC cell lines. **A** The knockdown efficiencies of various MEX3A shRNAs in C666-1 cells were determined by qRT-PCR. **B** MEX3A protein levels in shCtrl and shMEX3A stable C666-1 and HONE-1 cell lines were detected by western blotting. **C** Cell proliferation rates of C666-1 and HONE-1 cells after shCtrl and shMEX3A lentivirus infection were determined by MTT assay. **D** Colony formation abilities of C666-1 and HONE-1 cells infected with shCtrl or shMEX3A lentivirus were detected by colony formation assay. **E** Migration rates of C666-1 and HONE-1 cells were determined by wound healing assay at 0, 24, and 48 h after infection. **F** Migration and invasion abilities of C666-1 and HONE-1 cells infected with shCtrl and shMEX3A lentivirus were determined by Transwell assay. Magnification: 200×. **G** The effect of MEX3A knockdown on cell apoptosis was measured by flow cytometry. *P < 0.05, **P < 0.01, ***P < 0.001.

To explore the role of MEX3A in NPC in vitro, C666-1 and HONE-1 cells were infected with shCtrl and shMEX3A lentivirus particles. As shown in Fig. 2A, all three shMEX3A constructs tested reduced MEX3A mRNA expression in C666-1 cells. As shMEX3A-2 was the most effective, it was used for subsequent experiments. In addition, we confirmed sufficient lentivirus infection efficacy (Fig. S1A), effective knockdown of MEX3A mRNA (Fig. S1B) and MEX3A protein (Fig. 2B) levels in infected C666-1 and HONE-1 cells (*P* < 0.01 or 0.001).

We next explored the impact of MEX3A knockdown on the biological characteristics of NPC cells. MEX3A depletion significantly reduced the proliferation rates of C666-1 and HONE-1 cells (*P* < 0.001, Fig. 2C). Additionally, shMEX3A C666-1 and HONE-1 cells showed significantly attenuated colony formation abilities when compared with their shCtrl counterparts (*P* < 0.001, Fig. 2D). Furthermore, MEX3A depletion resulted in significant reductions in migration (*P* < 0.05, Fig. 2E) and invasion (*P* < 0.001, Fig. 2F) as indicated by wound-healing and Transwell assays, respectively. The apoptosis rate increased in both C666-1 and HONE-1 cells after infection with shMEX3A lentivirus as indicated by flow cytometry (*P* < 0.001, Fig. 2G). Taken together, these results indicated that MEX3 A knockdown leads to the inhibition of NPC cell proliferation and migration and promotion of apoptosis in vitro.

### MEX3A is involved in the tumorigenesis of NPC in vivo

**Fig. 3:**
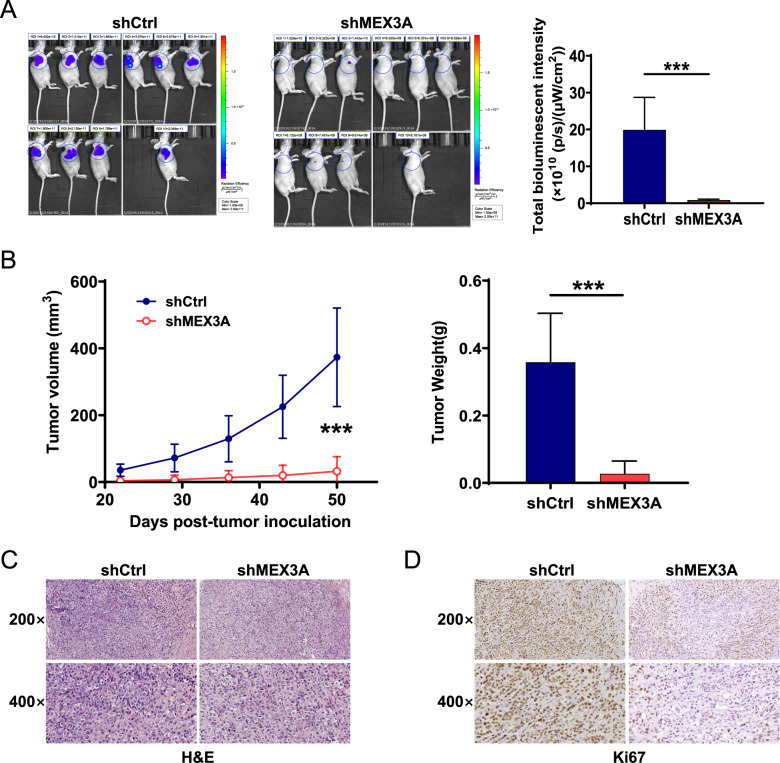
MEX3A knockdown suppresses tumorigenesis of NPC in vivo. **A** The fluorescence intensity of xenograft tumors was observed through injection of D-luciferin before sacrificing mice at the study endpoint. **B** Tumor volumes were calculated at the indicated days after tumor inoculation. **C**, **D** Representative images of H&E (**C**) and Ki-67 protein (**D**) staining of xenograft tumors from both groups. Magnification: 200× or 400×. ***P < 0.001.

We next investigated the involvement of MEX3A in NPC in vivo using a xenograft mouse model. In vivo imaging demonstrated significantly reduced fluorescence intensity in the shMEX3A group (*P* < 0.001, Fig. 3A), which indicated compromised tumor growth. Indeed, xenograft tumor volumes and weights were significantly lower in the shMEX3A group than in the shCtrl group (*P* < 0.001, Fig. 3B and S2). Furthermore, H&E (Fig. 3C) and Ki-67 immunohistochemical (Fig. 3D) staining revealed reduced proliferative activity upon MEX3A depletion. Together, these data suggested that MEX3A promotes tumor growth in vivo.

### SCIN is a downstream target of MEX3A in NPC

**Fig. 4:**
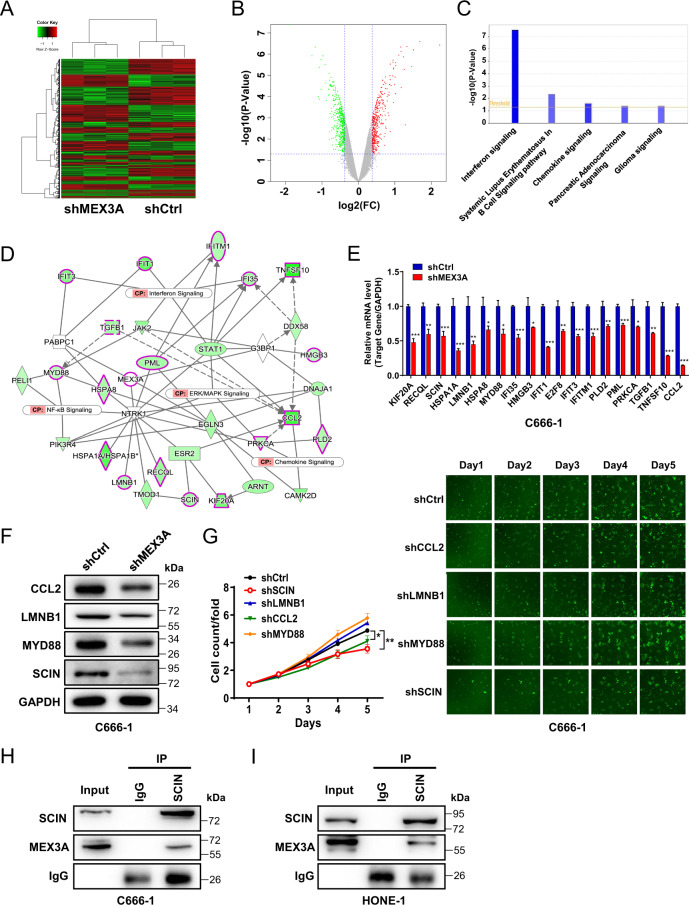
MEX3A interacts with SCIN in NPC cells. **A** Heatmap of DEGs identified by hierarchical clustering analysis of RNA-sequencing data of shCtrl (*n* = 3) and shMEX3A (*n* = 3) stable C666-1 cells. **B** Volcano plot of DEGs between shCtrl and shMEX3A stable C666-1 cells. **C** Classic pathway enrichment analysis revealed that MEX3A was associated with interferon and chemokine signaling. **D** Ingenuity pathway analysis-based interaction network of MEX3A and signaling pathways, including the interferon, chemokine, NF-κB, ERK/MAPK signaling pathways. **E**, **F** The expression levels of major downstream factors associated with MEX3A were determined by RT-qPCR (**E**) and western blotting (**F**) in C666-1 cells. **G** The impact of depletion of main downstream genes associated with MEX3A, including SCIN, CCL2, LMNB1, and MYD88, on the proliferation of C666-1 cells was determined by Celigo-based cell counting assay. **H**, **I** The interaction between MEX3A and SCIN in C666-1 cells (**H**) and HONE-1 cells (**I**) was determined by endogenous co-immunoprecipitation. *P < 0.05, **P < 0.01, ***P < 0.001.

Next, we examined the possible molecular mechanism(s) of MEX3A in NPC by identifying DEGs between shCtrl and shMEX3A C666-1 cells. We identified 427 upregulated and 434 downregulated DEGs after MEX3A knockdown (Fig. 4A, B and S3A, Table S10). Classic pathway enrichment analysis based on IPA suggested that the identified DEGs were significantly enriched in several diseases, including cancer, organismal injury and abnormalities, and gastrointestinal disease (Fig. S3B, C), and pathways including “interferon signaling”, “systemic lupus erythematosus in B cell signaling pathway”, and “chemokine signaling” (Z-score ≤ –2) (Fig. 4C and S4). An IPA-based interaction network between MEX3A and DEGs related to the significantly enriched signaling pathways and their related canonical pathways (interferon, chemokine, NF-κB, and ERK/MAPK signaling pathways) (Fig. 4D) revealed that CCL2, HMGB3, HSPA1A*/*HSPA1B, HSPA8, IFI35, IFIT1, IFIT3, IFITM1, KIF20A, LMNB1, MYD88, PLD2, PML, PRKCA, RECQL, SCIN, TGFB1, and TNFSF10 were potential downstream interactors of MEX3A. Compared with shCtrl C666-1 cells, shMEX3A C666-1 cells displayed significantly reduced CCL2, LMNB1, MYD88, and SCIN mRNA (Fig. 4E) and protein (Fig. 4F) levels. Moreover, among the main downstream genes associated with MEX3A, reduced SCIN expression was associated with significantly reduced cell proliferation rate (Fig. 4G). More importantly, the endogenous co-IP assay indicated a direct interaction between MEX3A and SCIN in C666-1 (Fig. 4H) and HONE-1 cells (Fig. 4I). Together, these data indicated that SCIN is a direct downstream target of MEX3A in NPC cells.

### SCIN is involved in MEX3A-induced regulation of NPC

**Fig. 5:**
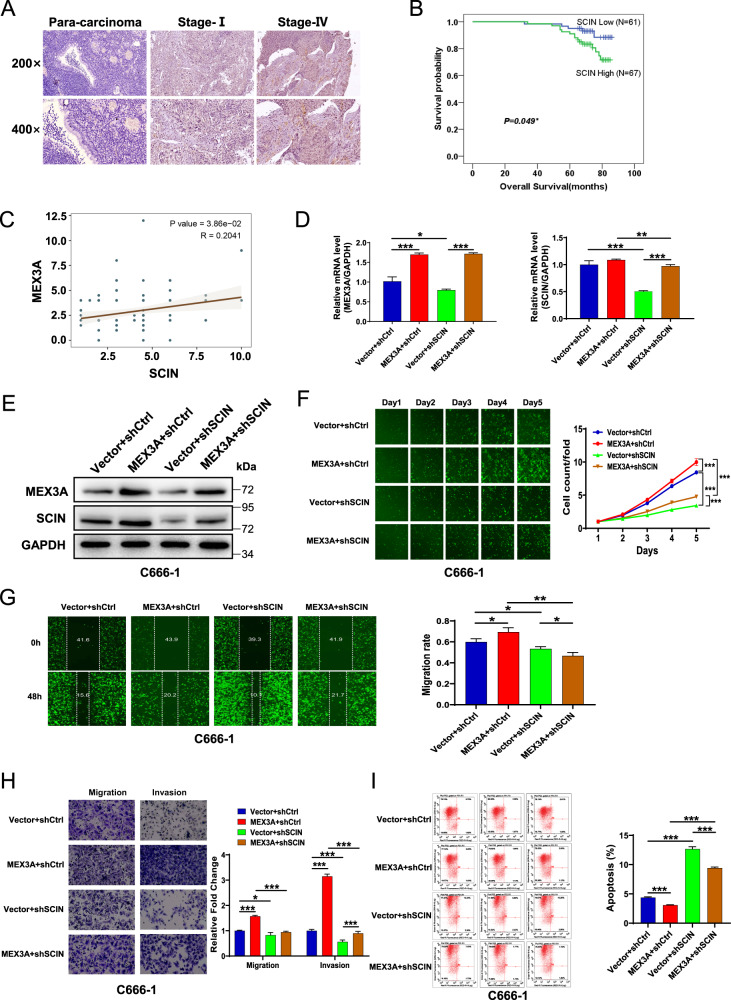
SCIN is involved in MEX3A-mediated regulation of NPC. **A** Compared with normal nasopharyngeal tissues, SCIN levels were higher in NPC tissues, especially in the advanced stage group. **B** Kaplan–Meier curves of overall survival of NPC patients with high or low SCIN expression (*n* = 128). **C** Immunohistochemical staining scores of SCIN was positively correlate with that of MEX3A in NPC TMAs. **D**, **E** Relative mRNA (**D**) and protein (**E**) levels of MEX3A and SCIN in four constructed stable cell lines were determined by RT-qPCR and western blotting, respectively. **F** The effects of MEX3A overexpression, SCIN knockdown, and simultaneous MEX3A overexpression plus SCIN knockdown on the proliferation of C666-1 cells were analyzed by Celigo-based cell counting assay. **G** The effects of MEX3A overexpression, SCIN knockdown, and simultaneous MEX3A overexpression plus SCIN knockdown on C666-1 cell migration were analyzed by wound-healing assay. **H** The effects of MEX3A overexpression, SCIN knockdown, and simultaneous MEX3A overexpression plus SCIN knockdown on C666-1 cell migration and invasion were determined by Transwell assay. **I** The effects of MEX3A overexpression, SCIN knockdown, and simultaneous MEX3A overexpression plus SCIN knockdown on the apoptosis rate of C666-1 cells were determined by flow cytometry. The results are presented as mean ± SD. *P < 0.05, **P < 0.01, ***P < 0.001.

We next examined SCIN expression in NPC TMAs (Table S1). SCIN levels in NPC tissues were significantly higher than those in non-cancerous tissues (*P* < 0.001, Fig. 5A, Table. 4S). Higher SCIN expression was correlated with advanced stage NPC and worse prognosis (*P* < 0.05, Fig. 5A-B, Table. 5S-6S). The expression level of SCIN was positively correlated with that of MEX3A according to their individual staining H-scores acquired from the NPC TMAs (*R* = 0.20, *P* < 0.05, Fig. 5C). Similar to the MEX3A expression pattern, C666-1 and HONE-1 NPC cells displayed high SCIN mRNA expression levels (Fig. S5A). Subsequently, we constructed (Fig. S5B) and confirmed the infection efficiency (Fig. S6) of four stable C666-1 cell lines: control (Vector+shCtrl), MEX3A overexpression (MEX3A + shCtrl), SCIN knockdown (Vector+shSCIN), and simultaneous MEX3A overexpression plus SCIN knockdown (MEX3A + shSCIN). RT-qPCR (Fig. 5D) and western blot analyses (Fig. 5E) confirmed effective infection and target manipulation. As expected, MEX3A overexpression increased the proliferative activity of the control and SCIN knockdown groups compared to their respective counterparts. More importantly, SCIN knockdown significantly inhibited cell proliferation and rescued the enhancing effect of MEX3A overexpression on cell proliferation (*P* < 0.001, Fig. 5F). Further, NPC cell migration and invasion (*P* < 0.05 or 0.001, Fig. 5G-H) were significantly reduced upon SCIN knockdown in both the control and MEX3A groups. Moreover, MEX3A overexpression resulted in a significantly decreased apoptosis rate, which was rescued by SCIN depletion in C666-1 cells (*P* < 0.01 or 0.001, Fig. 5I). Together, these results supported the carcinogenic role of MEX3A in NPC, which was attenuated upon SCIN knockdown, suggesting that SCIN is a downstream regulator in MEX3A-induced NPC.

### hsa-miRNA-3163 is involved in MAX3A-mediated regulation of SCIN in NPC

**Fig. 6:**
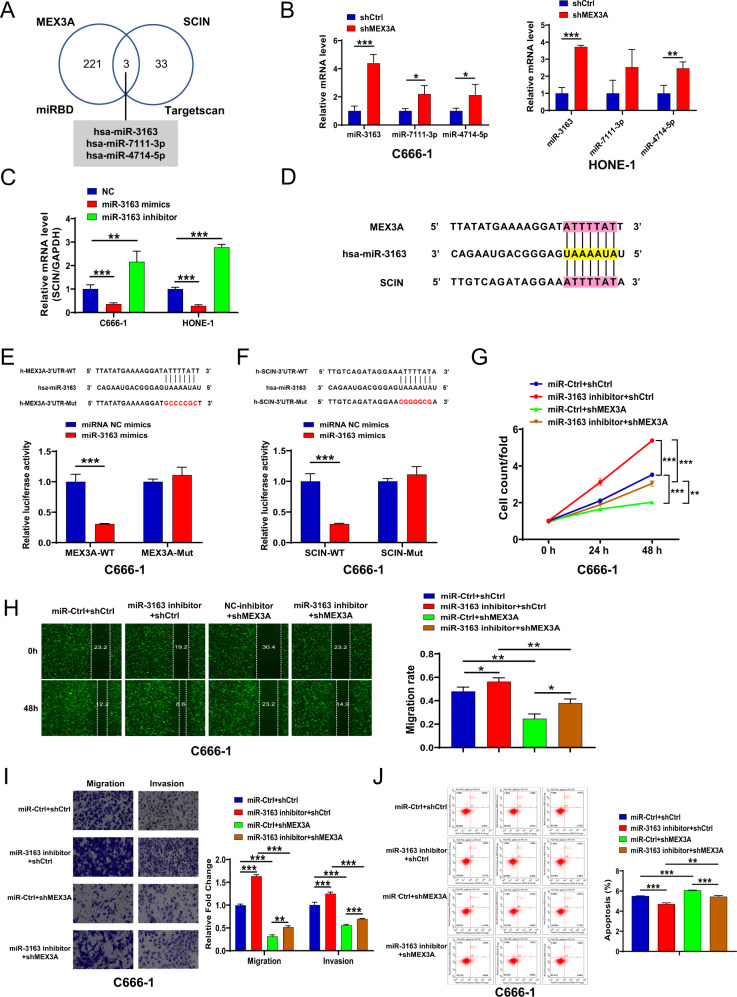
has-miRNA-3163 was involved in MAX3A mediated regulation of SCIN in nasopharyngeal carcinoma. **A** The intersected miRNAs were predicted from two datasets (miRDB and Targetscan). **B** Indicated miRNAs levels in shCtrl and shMEX3A stable C666-1 and HONE-1 cell lines were determined by RT-qPCR. **C** SCIN mRNA levels were detected by RT-qPCR after the negative control (NC), miRNA-3163 mimics or miRNA-3163 inhibitor transfected into C666-1 and HONE-1 cells. **D** A schematic diagram representing the predicted binding sites for miRNA-3163, MEX3A and SCIN. **E** Direct binding between miRNA-3163 and MEX3A was assessed by dual-luciferase assay after C666-1 cells were co-transfected with the NC, miRNA-3163 mimics, and wild-type (WT) or mutant (Mut) MEX3A luciferase reporter vectors. **F** Direct binding between miRNA-3163 and SCIN was assessed by dual-luciferase assay. **G** The effects of miRNA-3163 inhibition, MEX3A knockdown, and simultaneous MEX3A knockdown plus miRNA-3163 inhibition on the proliferation rates of C666-1 cells were determined by CCK-8 assay. **H**, **I** Wound healing and transwell assays were conducted to assess the migration and invasion abilities of cells under the indicated treatments. **J** The apoptosis rates of C666-1 cells under the indicated treatments were analyzed by flow cytometry. The results are presented as mean ± SD. *P < 0.05, **P < 0.01, ***P < 0.001.

Next, we explored the underlying regulatory mechanism between MEX3A and SCIN. Emerging studies have reported the biological function of miRNAs in tumorigenesis. Therefore, we want to investigate the potential miRNAs that involved in MEX3A-SCIN axis in NPC. Analysis of the miRDB (http://mirdb.org/mirdb/index.html) and Targetscan (http://www.targetscan.org/vert_72/) datasets indicated that three miRNAs were predicted to interact with both MEX3A and SCIN (Fig. 6A). Considering that decreased hsa-miRNA-3163 expression was previously reported in NPC tissues [26], we selected hsa-miRNA-3163 for further study. We also verified the MEX3A knockdown led to increased expression of miRNA-3163 in NPC cells (*P* < 0.001, Fig. 6B). Furthermore, SCIN was downregulated in NPC cells transfecting with miRNA-3163 mimics, but was upregulated in the miRNA-3163 inhibitors group (*P* < 0.001, Fig. 6C). Thus, we speculated that has-miRNA-3163 might involve in MEX3A-SCIN axis in NPC cells.

The prediction from miRDB and Targetscan datasets indicates that MEX3A and SCIN may binds to the same site in hsa-miRNA-3163 (Fig. 6D). The dual-luciferase reporter system was utilized to verify their binding sites with miRNA-3163 (Fig. 6E, F). After co-transfection with the miRNA-3163 mimics, the relative luciferase activities of WT SCIN, but not Mut SCIN were significantly reduced (*P* < 0.001, Fig. 6E), indicating direct binding between miRNA-3163 and the SCIN 3’-UTR region. Dual-luciferase reporter assay also indicated the direct binding of MEX3A 3’-UTR with miRNA-3163 (*P* < 0.001, Fig. 6F). More importantly, transfection with the miRNA-3163 inhibitor rescued the tumor suppressive effects of MEX3A knockdown in NPC cells (*P* < 0.05, 0.01 or 0.001, Fig. 6G–J), indicating that miRNA-3163 is a downstream regulator of MEX3A.

### The MEX3A-SCIN axis regulates NPC likely via NF-κB signaling

**Fig. 7:**
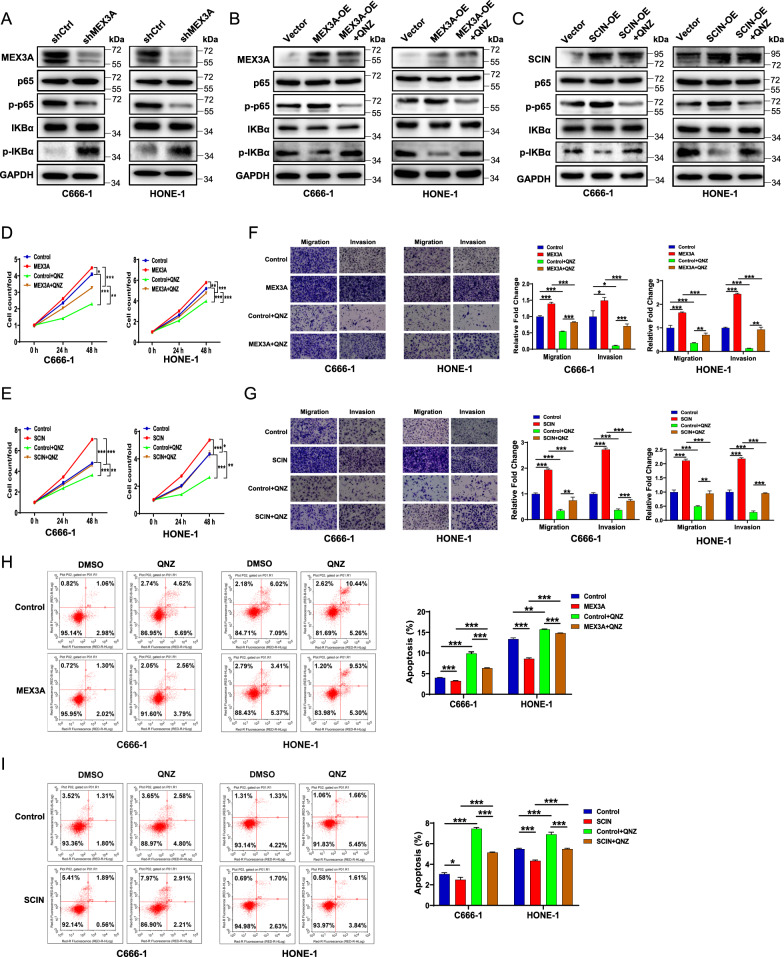
MEX3A and SCIN regulate NPC likely via the NF-κB signaling pathway. **A** The major components of the NF-κB pathway in shCtrl and shMEX3A stable C666-1 and HONE-1 cell lines were detected by western blotting. **B**, **C** The effects of treatment with NF-κB inhibitor QNZ (EVP4593) on MEX3A (**B**) or SCIN (**C**) overexpression in human C666-1 and HONE-1 NPC cells were determined by western blotting. **D**, **E** The effects of QNZ compared with those of negative control (DMSO) on the proliferation of C666-1 and HONE-1 cells with or without MEX3A (**D**) or SCIN (**E**) overexpression were detected by CCK-8 assay. **F**, **G** The impact of QNZ on the migration and invasion abilities of indicated cells with or without MEX3A (**F**) or SCIN (**G**) overexpression was measured by Transwell assay. **H**, **I** The impact of QNZ on the apoptosis rates of indicated cells with or without MEX3A (**F**) or SCIN (**G**) overexpression was measured by flow cytometry. The results are presented as mean ± SD. *P < 0.05, **P < 0.01, ***P < 0.001.

As mentioned above, IPA suggested that interferon and NF-κB signaling were the most likely downstream pathways underlying MEX3A-SCIN axis-mediated regulation of NPC. Western blot analysis of the major NF-κB pathway components after MEX3A knockdown in both C666-1 and HONE-1 cells revealed significant downregulation of p-p65, which indicated decreased NF-κB signaling activity (Fig. 7A). We further constructed stable MEX3A- or SCIN-overexpressing C666-1 and HONE-1 cell lines. Unsurprisingly, overexpression of MEX3A or SCIN led to increased NF-κB signaling activity in both NPC cell lines (Fig. 7B, C). Notably, treatment with NF-κB activation inhibitor QNZ (EVP4593) mitigated the promotive effect of MEX3A (Fig. 7B) or SCIN (Fig. 7C), supporting the involvement of NF-κB signaling in MEX3A-SCIN axis-mediated NPC.

Furthermore, we explored whether the NF-κB activation inhibitor could alleviate the oncogenic effects of MEX3A and SCIN in NPC cells. MEX3A overexpression in C666-1 and HONE-1 cells increased cell proliferation (*P* < 0.01 or 0.001, Fig. 7D), migration, and invasion (*P* < 0.05 or 0.001, Fig. 7F), but decreased apoptosis (*P* < 0.001, Fig. 7H), all of which were reversed by QNZ treatment (*P* < 0.05, 0.01 or 0.001, Fig. 7D, F, H). The similar phenotype was also observed upon SCIN overexpression in NPC cells (*P* < 0.05, 0.01, or 0.001, Fig. 7E, G, I), indicating that NF-κB signaling is a downstream regulator of the MEX3A-SCIN axis in NPC cells.

## Discussion

The results of this study demonstrated that MEX3A is highly expressed in NPC tissues. Elevated MEX3A levels were observed more frequently in advanced stage than in early stage NPC. In addition, high MEX3A expression was significantly associated with worse overall survival. Depletion of MEX3A in NPC cells led to decreased proliferation, colony formation, and migration, as well as increased apoptosis. Furthermore, the oncogenic role of MEX3A was demonstrated in an NPC xenograft mouse model. These results support that MEX3A may serve as an independent biomarker in NPC development. To the best of our knowledge, this study is the first to associate MEX3A expression with NPC progression.

Bioinformatics analysis of TCGA data previously revealed that MEX3A somatic copy number and expression are upregulated in multiple cancer types [10]. However, relatively little is known about the oncogenic functions and downstream molecular targets of MEX3A in malignancies [15, 18]. Several studies have suggested the involvement of PI3K-Akt signaling in MEX3A-mediated carcinogenesis [15, 17, 27]. Li et al. reported that MEX3A promotes colorectal cancer development via the RAP1/MAPK pathway [28]. Panzeri et al. revealed that MEX3A binds and promotes the stability of CDK6 mRNA, affecting the efficiency of chemotherapy in pancreatic ductal adenocarcinoma [11]. Moreover, MEX3A has been suggested to promote tumor development in glioma by targeting CCL2 [18]. Thus, the molecular mechanisms by which MEX3A mediates tumorigenesis are complex and same to be tissue-dependent.

In this study, whole-transcript expression array analysis and IPA revealed SCIN to be a potential downstream target of MEX3A in NPC cells. Furthermore, we verified the direct interaction between SCIN and MEX3A through co-IP analysis. More importantly, the oncogenic effects of MEX3A overexpression in NPC cells, including increased cell proliferation, migration, and invasion but decreased apoptosis, were all rescued upon SCIN depletion. Taken together, these data suggest that SCIN is a potential downstream target of MEX3A. First identified as a calcium-dependent F-actin-severing protein in chromaffin cells [29], SCIN regulates actin cytoskeleton dynamics and is involved in numerous actin-related processes [20, 29, 30]. SCIN overexpression is commonly observed in several types of malignancies [21, 22, 31], but is not correlated with prognosis in head and neck cancers [32]. Interestingly, SCIN overexpression inhibited tumor proliferation in vitro and tumorigenesis in vivo in megakaryoblastic leukemia cells [20], with similar effects reported in another type of leukemia [33]. Thus, similar to MEX3A, the role of SCIN in tumorigenesis seems to be context- and tissue-dependent.

Emerging evidence indicates that miRNA-3163 is downregulated and functions as a tumor suppressor in various cancers [26, 34, 35]. A previous study reported that miRNA-3163 exerted its tumor suppression function by downregulating TWIST-1 in NPC [35]. Our present study suggests the involvement of miRNA-3163 in the regulation of the MEX3A-SCIN axis. Considering one miRNA may exert its function through different pathways, our work extends the current knowledge of miRNA-3163 functions in NPC.

The bioinformatics analysis undertaken in the current study suggested that NF-κB signaling is one of the key downstream pathways of MEX3A in NPC cells. Concurring with these findings, a previous study revealed that aberrant NF-κB signaling led to NPC development and progression [1]. Measuring the major NF-κB signaling-related proteins after MEX3A knockdown indicated decreased levels of phosphorylated p65, a marker of NF-κB signaling activity [36]. Moreover, treatment with an NF-κB signaling-specific inhibitor reversed the pro-oncogenic effects of MEX3A or SCIN overexpression in NPC cells, further suggesting that the NF-κB signaling pathway may be a downstream effector of the MEX3A/SCIN axis in NPC.

In conclusion, the results of this study demonstrate that MEX3A acts as an oncoprotein in NPC. Specifically, miRNA-3163, SCIN, and the NF-κB pathway are potential downstream targets involved in MEX3A-mediated tumorigenesis in NPC. This novel signaling network may serve as a clinical prognostic and therapeutic biomarker for NPC treatment.

## Supplementary information

supplementary materials

aj-checklist

## Data Availability

The data that support the findings of this study are available from the corresponding author upon reasonable request.
