# Supplementary material for: MEX3A promotes nasopharyngeal carcinoma progression via the miR-3163/SCIN axis by regulating NF-κB signaling pathway
Source: Cell Death Dis. 2022 Apr 30;13(4):420. doi: 10.1038/s41419-022-04871-0 (PMC9056523; doi:10.1038/s41419-022-04871-0)
Supplement: Supplementary file 1 — supplementary materials [file 41419_2022_4871_MOESM1_ESM.pdf]

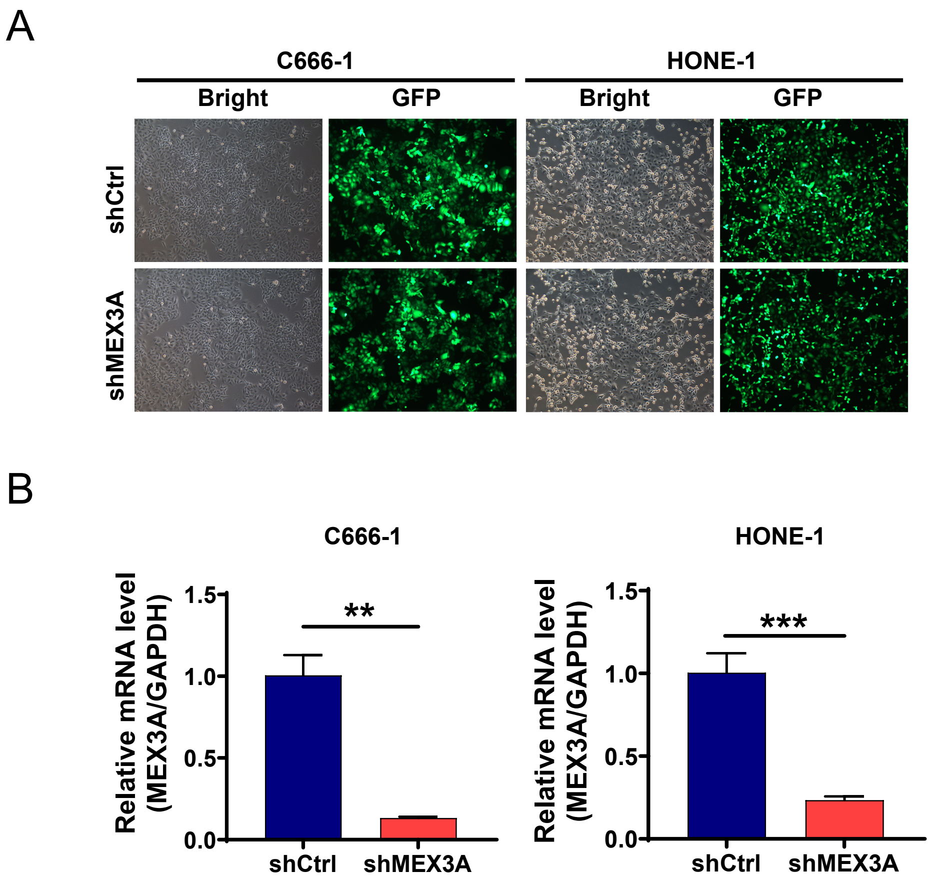

Figure S1. The infection efficiencies of shMEX3A and shCtrl in C666-1 and HONE-1 nasopharyngeal cancer cell lines were evaluated by observing GFP fluorescence (A) and mRNA levels (B). $^{**}P < 0.01$, $^{***}P < 0.001$

shCtrl

shMEX3A

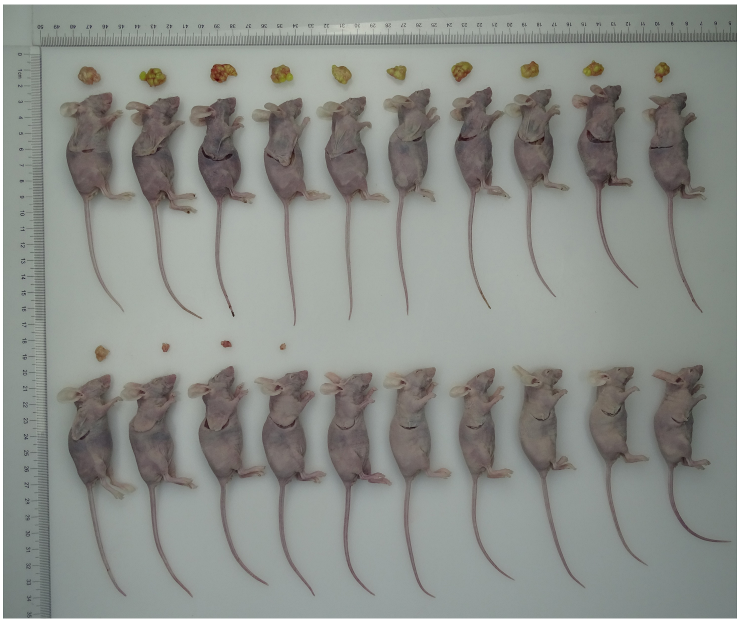

Figure S2. Photographs of xenograft tumors from shCtrl and shMEX3A groups.

A

B

C

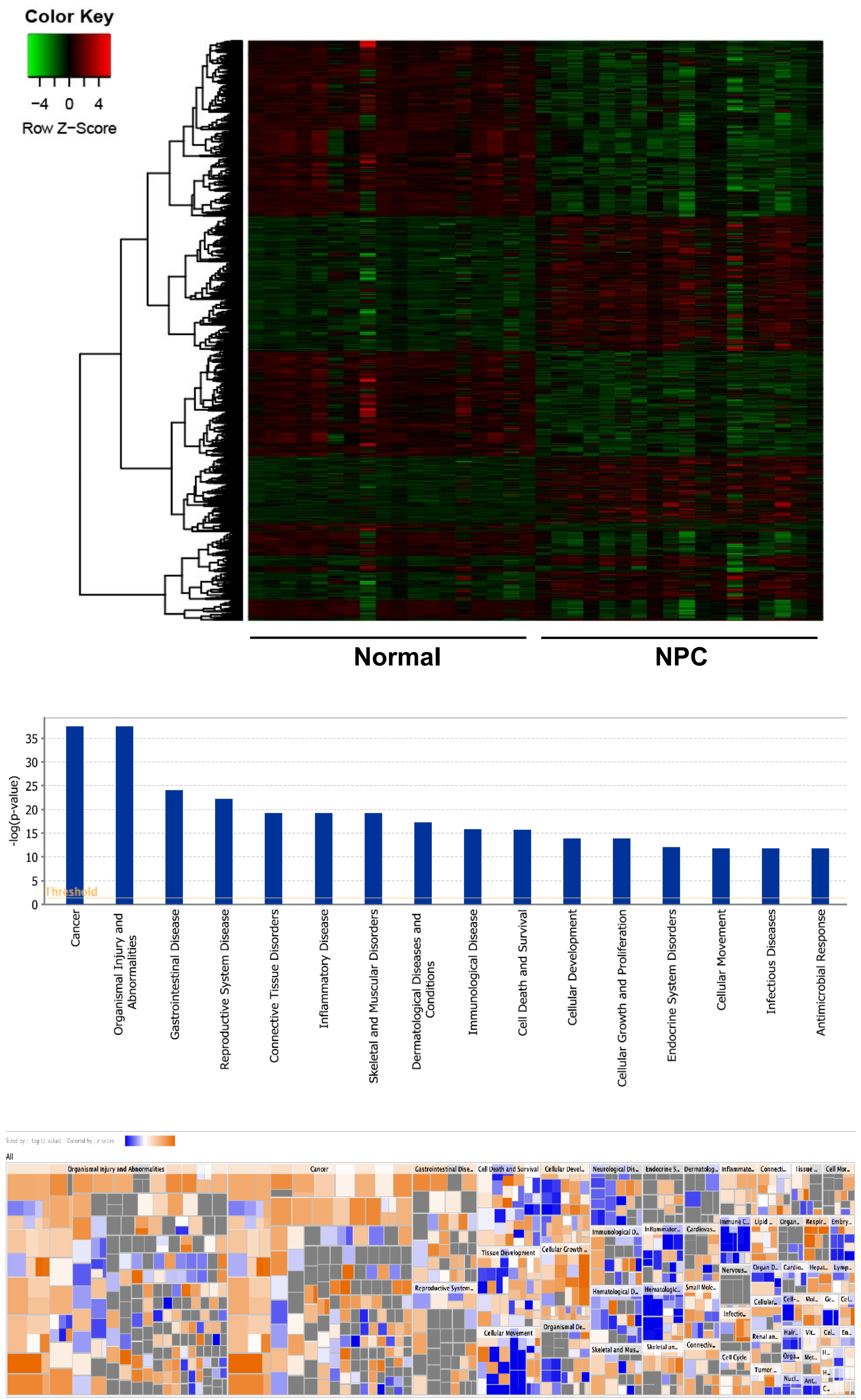

Figure S3. (A) The heat map of gene expression profiling difference between nasopharyngeal carcinoma tissues and normal tissues according to data in GSE53819. (B) The enrichment of the DEGs in IPA disease. (C)The function of the DEGs was analyzed by IPA.

A

Upstream Regu...	Expr Log Ratio	Molecule Type	Predicted Activ...	Activation z-sc...	p-value of ...	Target Molecul...	Mechanistic Ne...
lipopolysaccharide		chemical drug		-0.117	6.35E-25	↑ACKR3, ↑... ...all 161	323 (16)
MAPK1		kinase	Activated	5.149	1.11E-23	↓ATP1B1,↓B......all 60	304 (21)
dexamethasone		chemical drug		1.020	1.38E-23	↑ACKR3, ↑... ...all 168	337 (16)
IFNL1		cytokine	Inhibited	-4.893	2.77E-23	↓C19orf66,↑......all 31	281 (16)
IFNA2		cytokine	Inhibited	-6.222	3.70E-23	↓C19orf66,↓......all 49	170 (16)
IFNG		cytokine	Inhibited	-3.172	1.78E-22	↑ADM, ↑AL... ...all 128	297 (18)
beta-estradiol		chemical - endogenou...		1.225	2.46E-22	↑ACKR3, ↑... ...all 159	359 (23)
tretinoin		chemical - endogenou...	Inhibited	-2.726	4.74E-22	↓ACSS2,↑A......all 138	315 (17)
Interferon alpha		group	Inhibited	-3.944	1.43E-20	↓APOL2,↓BC......all 63	259 (16)
TNF		cytokine		0.788	5.32E-20	↑ACKR3, ↑... ...all 147	286 (15)
TGM2		enzyme	Inhibited	-4.073	2.62E-19	↓ACSS2,↑AQ......all 43	315 (23)
IL1B	↑0.816	cytokine		0.869	1.20E-18	↑ADAMTS1,↑......all 93	282 (13)
TGFB1	↓-0.516	growth factor		1.346	6.42E-18	↑ACSL3,↑A......all 140	321 (20)
STAT3		transcription regulator		1.277	7.03E-18	↑ADM, ↑AREG,...all 69	319 (21)
poly rI:rC-RNA		biologic drug		-1.076	8.02E-18	↑ACSL3,↑BM......all 65	307 (18)
PRL		cytokine	Inhibited	-3.944	9.92E-18	↓BCL6,↓C19......all 46	283 (22)
IRF1		transcription regulator	Inhibited	-3.399	8.82E-17	↓C1R, ↓CCL2, ...all 36	261 (17)
progesterone		chemical - endogenou...		0.692	3.42E-16	↑ADAMTS1,↑......all 68	303 (18)
IRF7		transcription regulator	Inhibited	-5.118	3.82E-16	↓DDX58,↓D... ...all 34	192 (18)
IL1RN		cytokine		1.663	1.90E-15	↓BTN3A1, ↓... ...all 29	283 (17)
STAT1	↓-0.513	transcription regulator	Inhibited	-3.661	2.36E-15	↓BCL6, ↓C1R, ...all 49	284 (20)
fluticasone propionate		chemical drug		0.613	3.73E-15	↑ADM, ↑AQP9,...all 33	264 (17)
PDGF BB		complex	Activated	2.267	4.15E-15	↑ACKR3, ↓A... ...all 46	280 (21)
IFNB1		cytokine	Inhibited	-3.551	2.49E-14	↓APOL2,↓BT......all 42	249 (17)
ESR1		ligand-dependent nuc...		-0.098	2.91E-14	↑ACKR3, ↓... ...all 105	352 (22)
IFNAR2		transmembrane recep...	Inhibited	-2.121	1.75E-13	↓DDX58,↓IF... ...all 17	248 (13)
RNASEH2A		enzyme			3.33E-13	↑ACKR3, ↓C... ...all 16	

B

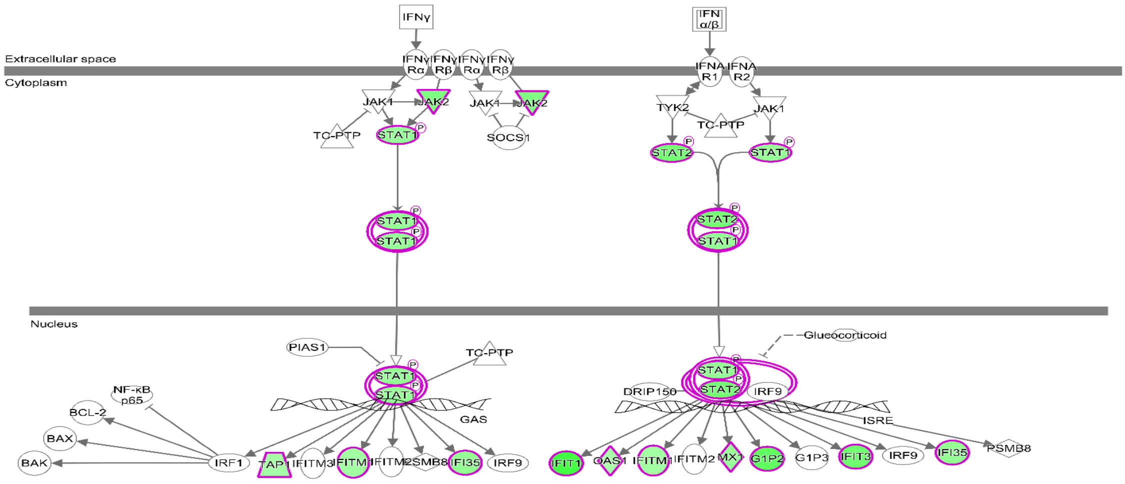

C

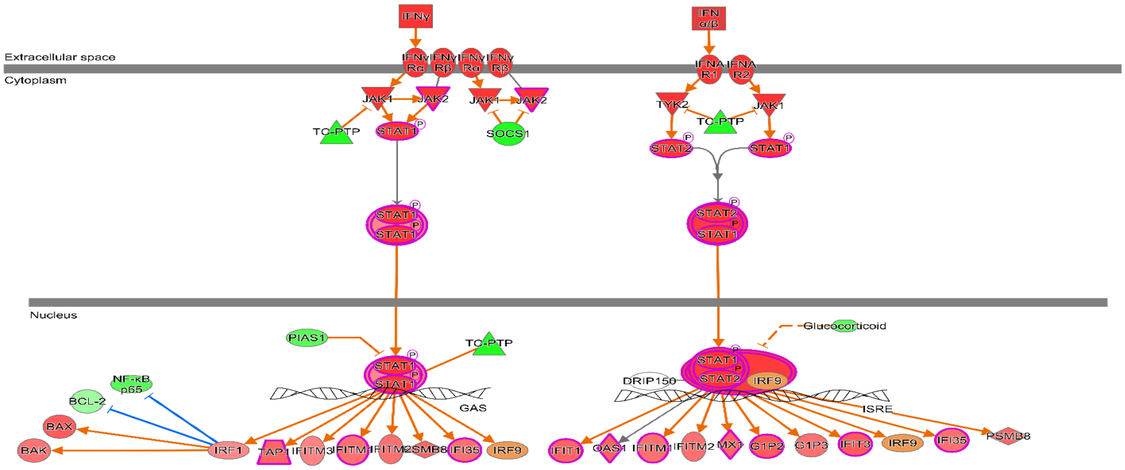

Figure S4. (A) The upstream regulators that associated with DEGs identified by transcription array. (B) The enrichment of the DEGs in canonical signaling pathways was analyzed by IPA. (C) The proposed regulation of indicated DEGs when interferon signaling was activated. The molecules labeled in green indicates downregulated molecules, while in red indicates upregulated molecules.

A

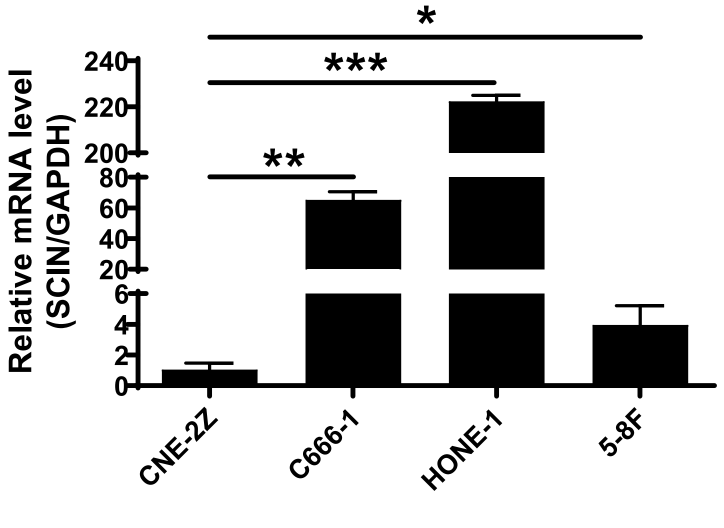

B

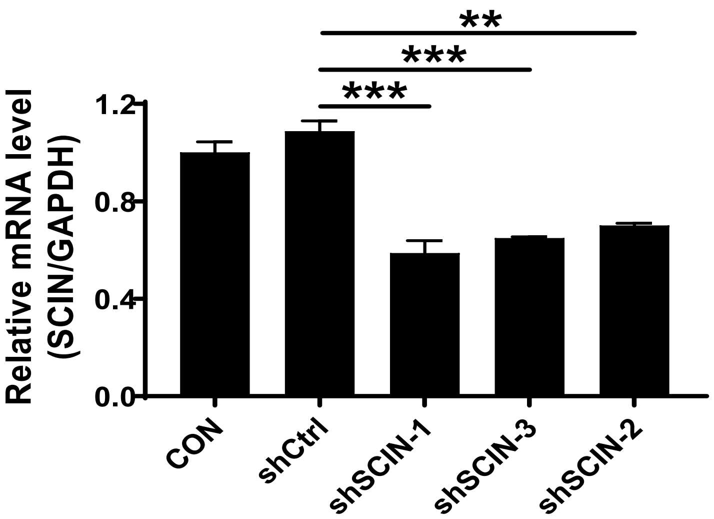

Figure S5. (A) The knockdown efficiencies of three SCIN shRNA were evaluated by qRT-PCR. (B) The internal mRNA expression levels of indicated nasopharyngeal carcinoma cell lines.

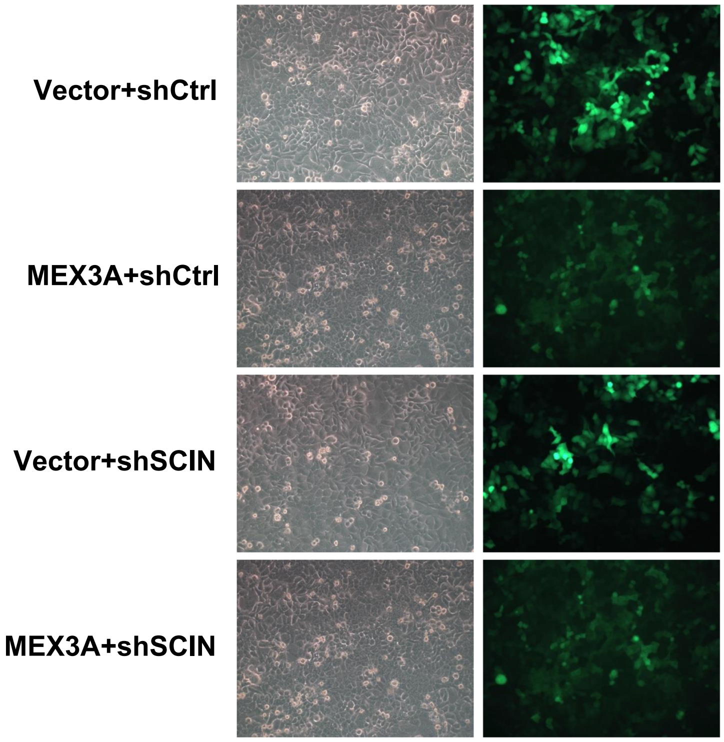

Figure S6. The infection efficiencies of indicated C666-1 stable cell lines were evaluated through observing GFP fluorescence.

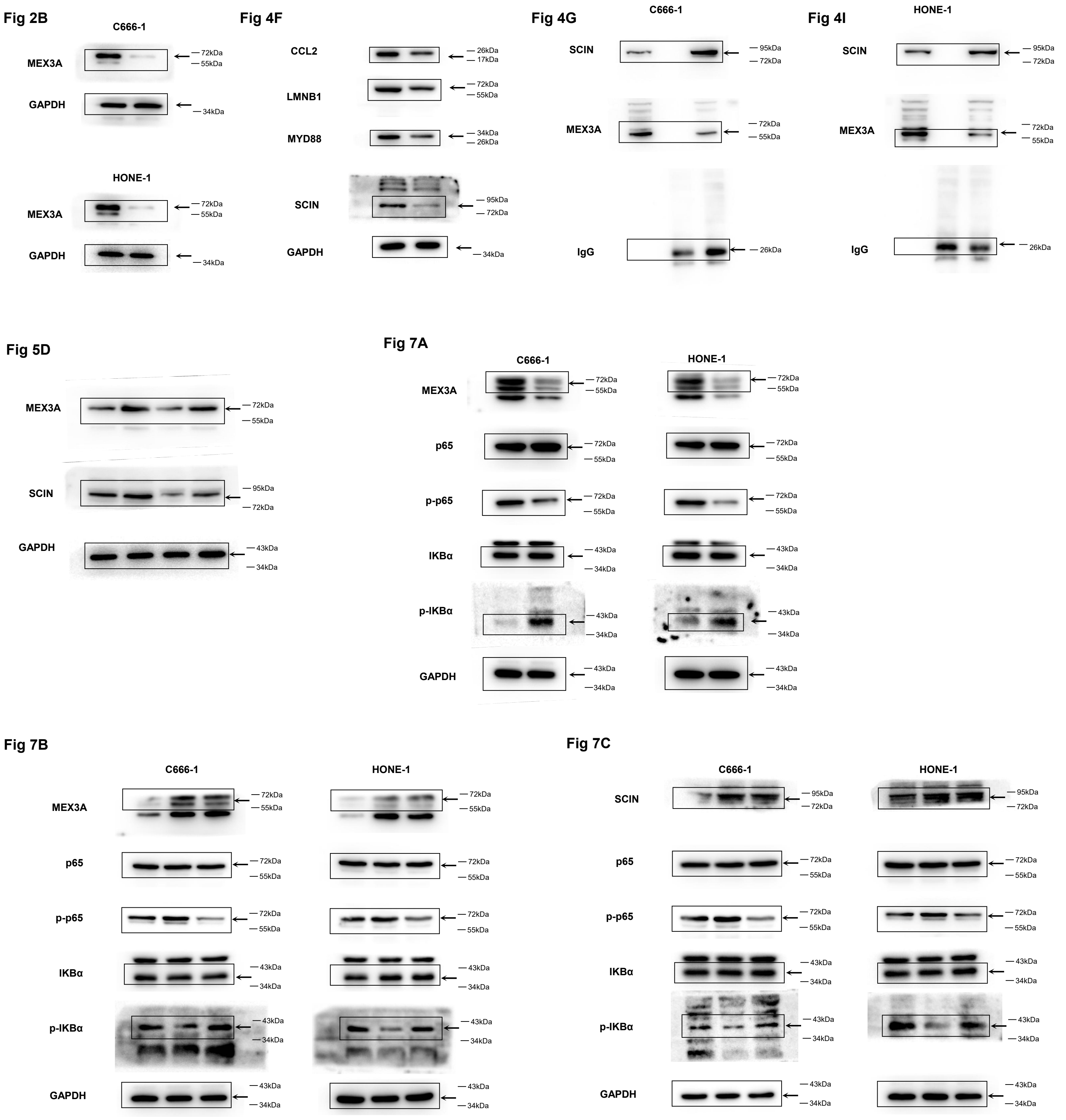

Figure S7. The uncropped western blotting image of indicated figure panels

Table S1. MEX3A Expression patterns in nasopharyngeal carcinoma tissues and para-carcinoma tissues revealed in immunohistochemistry analysis

MEX3A expression	Tumor tissue		Para-carcinoma tissue		*P* value
	Cases	Percentage	Cases	Percentage	
Low	59	56.2%	28	93.3%	<0.001
High	46	43.8%	2	6.7%	

Table S2. Relationship between MEX3A expression and tumor characteristics in patients with nasopharyngeal carcinoma

Features	No. of patients	MEX3A expression		*P* value
		low	high	
All patients	105	59	46	
Age (years)				0.068
<48	51	24	27	
≥48	54	35	19	
Gender				0.037*
Male	79	49	30	
Female	26	10	16	
Tumor size				0.490
< 1.2cm	43	25	18	
≥1.2cm	51	26	25	
Stage				0.015*
I	15	9	6	
II	46	32	14	
III	28	13	15	
IV	16	5	11	
Recurrence				0.148
no	58	36	22	
yes	46	22	24	
Cervical lymph node metastasis				0.300
no	33	21	12	
yes	72	38	34	

$^*P < 0.05$

Table S3. Relationship between MEX3A expression and tumor characteristics in patients with nasopharyngeal carcinoma

MEX3A		*P*-value
Stage	Pearson correlation	0.238
	Significance (double tailed)	0.015*
	N	105
Gender	Pearson correlation	0.205
	Significance (double tailed)	0.036*
	N	105

*$P < 0.05$

Table S4. Expression patterns of SCIN in nasopharyngeal carcinoma tissues and para-carcinoma tissues revealed in immunohistochemistry analysis

SCIN expression	Tumor tissue		Para-carcinoma tissue		*P* value
	Cases	Percentage	Cases	Percentage	
Low	61	47.6%	9	90%	<0.001
High	67	52.4%	1	10%	

Table S5. Relationship between SCIN expression and tumor characteristics in patients with nasopharyngeal carcinoma

Features	No. of patients	SCIN expression		*P* value
		low	high	
All patients	128	61	67	
Age (years)				0.017*
<48	66	24	42	
≥48	62	37	25	
Gender				0.235
Male	99	50	49	
Female	29	11	18	
Tumor size				0.018*
< 1.2cm	56	36	20	
≥1.2cm	72	25	47	
Stage				0.000***
I	15	14	1	
II	55	38	17	
III	38	6	32	
IV	20	3	17	
Recurrence				0.012*
no	69	40	29	
yes	59	21	38	
Cervical lymph node metastasis				0.000***
no	36	28	8	
yes	92	33	59	

*$P < 0.05$, **$P < 0.01$, ***$P < 0.001$

Table S6. Relationship between SCIN expression and tumor characteristics in patients with nasopharyngeal carcinoma

**SCIN**		***P*-value**
Tumor size	Pearson correlation	0.209*
	Significance (double tailed)	0.018*
	N	128
Stage	Pearson correlation	0.582**
	Significance (double tailed)	0.000***
	N	128
Age (years)	Pearson correlation	0.213*
	Significance (double tailed)	0.017*
	N	128
Recurrence time	Pearson correlation	0.223*
	Significance (double tailed)	0.012*
	N	128
Cervical lymph node metastasis	Pearson correlation	0.377**
	Significance (double tailed)	0.000***
	N	128

*$P < 0.05$, **$P < 0.01$, ***$P < 0.001$

Table S7. Antibodies and reagents used in western blotting, IHC and co-IP

Antibodies used in western blotting

Primary antibodies	Dilution in WB	Source species	Company	Catalog No.
MEX3A	1:1000	Rabbit	Abcam	ab79046
CCL2	1:1000	Rabbit	Abcam	ab25124
LMNB1	1:4000	Rabbit	Shanying	12987-1-AP
MYD88	1: 1000	Rabbit	Cell signaling	4283
SCIN	1:1000	Rabbit	Abcam	ab199723
GAPDH	1:3000	Rabbit	Bioworld	AP0063
MEX3A	1:1000	Rabbit	Biorbyt	orb423033
P65	1:2000	Rabbit	Proteintech	10745-1-AP
p-P65	1:1000	Rabbit	CST	3033
IKBα	1:2000	Rabbit	Abcam	ab7217
p-IKBα	1:1000	Rabbit	Abcam	ab133462
SCIN	1:1000	Rabbit	Proteintech	11579-1-AP

Antibodies used in IHC

Primary antibodies	Dilution in IHC	Source species	Company	Catalog No.
Ki67	1:200	Rabbit	Abcam	ab16667
MEX3A	1:200	Rabbit	Abcam	ab79046
SCIN	1:1000	Rabbit	BIOSS	bs-1327R

Antibodies used in co-IP

Primary antibodies	Dilution in co-IP	Source species	Company	Catalog No.
SCIN	1:50/1:1000	Rabbit	Abcam	ab199723
MEX3A	1:1000	Rabbit	Abcam	ab79046

Secondary antibody

Secondary antibody	Dilution	Company	Catalog No.
HRP Goat Anti-Rabbit IgG (WB)	1:3000	Beyotime	A0208
HRP Goat Anti-Mouse IgG (WB)	1:3000	Beyotime	A0216
HRP Goat Anti-Rabbit IgG (IHC)	1:400	Abcam	ab6721
HRP Goat Anti-Rabbit IgG (co-IP)	1:3000	Beyotime	A0208

Table S8. The targeted sequences of shRNA

Gene	No.	Target sequence (5'-3')
MEX3A	1	ATAGCCGCTACTCCGACGCCT
MEX3A	2	AGGCAAGGCTGCAAGATTAAG
MEX3A	3	AGCCGCTCCAGGGCTTCTCTA
SCIN	1	CACGAGATGAGCTGACAACAT
SCIN	2	ATGAAGACAGCTGAAGAATTT
SCIN	3	GATGATTTAGCTGAAGATGAT

Table S9. Primers used in qPCR

Gene	Forward primer sequence (5'-3')	Reverse primer sequence (5'-3')
MEX3A	CGGAGTGGACTCTGGCTTTGAG	CAGAGGAGAAGAGCACGGAGGT
KIF20A	GGCCGTTCCTGCATGATTGT	TGTCTGCCTTAGCCCCTTTCT
RECQL	TAACAATGGCTGGAAAGGAGG	ATCAATGGGCAAATGACGAG
SCIN	ACAACATCTGCGTTCCTGACTG	GCTCTTTGCCTTGGGAGACT
HSPA1A	AGCTGGAGCAGGTGTGTAAC	CAGCAATCTTGGAAAGGCCC
LMNB1	CCCAGTTGGAAGCCTCCTTA	GCGAAACTCCAAGTCCTCAG
HSPA8	CCCGAGGTGTTCCTCAGATT	CGGCCCTTGTCATTAGTGATAGTA
MYD88	TTACAGGTGGCCGCTGTAGA	GGGGCAATAGCAGATGAAGG
IFI35	CTTCTTTGGCAAGACTAGGAACG	CGATTTGGCACAGACGCTGA
HMGB3	ACCGTCTGGATTCTTCCTGTT	GATGTAAGGCTGCTTTTCACTG
IFIT1	TGAAAAGGTGCTTGAAGTGGAC	GGGGAAGCAAAGAAAATGGC
E2F8	TGACGAAGTGGCAGAGGAAC	CATCATAATCTGCTCGGCGTA
IFIT3	CTCCTTGCCAAACAGATGTCC	CCTTGTAGCAGCACCCAATC
IFITM1	CGTGAAGTCTAGGGACAGGAAG	GCCGAATACCAGTAACAGGATGA
PLD2	TGTGATTCTTGGAGCAAATACCCG	GCCATGTCTTGCCACAACTGGA
PML	ACCCGCAAGACCAACAACA	TGGAACATCCTCGGCAGTAG
PRKCA	CTTCAGACAAAGACCGACGAC	CATCAGCTCCGAAACTCCAA
TGFB1	CAATTCCTGGCGATACCTCA	AAGGCGAAAGCCCTCAAT
TNFSF10	CTTACGTGTACTTTACCAACGAGC	GGGTCCCAATAACTGTCATCTT
CCL2	TAGAAGAATCACCAGCAGCAAG	CTTCGGAGTTTGGGTTTGC
GAPDH	TGACTTCAACAGCGACACCCA	CACCCTGTTGCTGTAGCCAAA
miR-3163	ACGATGGTATAAAATGAGGGCAG	GTGCAGGGTCCGAGGT
miR-4714-5p	TCCTGGAACTCTGACCCCTTAG	GTGCAGGGTCCGAGGT
miR-7111-3p	CCTTGATTATCCTCTCTTCCCTCC	GTGCAGGGTCCGAGGT
GAPDH	TGACTTCAACAGCGACACCCA	CACCCTGTTGCTGTAGCCAAA

Table S10. List of significantly downregulated transcripts upon shMEX3A

Genesymbol	logFC	P-value
RANBP3L	-1.91141894	3.98E-08
TNFSF10	-1.2736588	1.21E-06
LGALS9	-1.18495884	4.70E-07
CCL2	-1.1019837	9.91E-05
CROT	-1.07919475	0.000129855
TSPAN3	-1.07085963	4.23E-06
PTGS1	-1.02870843	1.83E-06
GLCE	-1.02260817	4.59E-05
HSPA1A	-0.97283718	1.20E-06
IFIT1	-0.91568034	3.07E-06
LAMP3	-0.89354184	2.28E-05
LGALS9C	-0.88134026	0.000150353
CUBN	-0.82089435	8.73E-05
TRIM22	-0.81461031	4.72E-05
RASL11A	-0.81081478	4.53E-05
MX2	-0.80383959	7.93E-05
RDH10	-0.80203495	8.00E-06
EHF	-0.79815761	1.69E-05
SPTLC3	-0.79520963	0.004531751
LYSMD2	-0.79373567	0.010246535
OAS2	-0.78606887	3.00E-05
LRIG1	-0.78164883	2.79E-05
PELI2	-0.77661719	0.000627126
UBA7	-0.76578472	2.79E-05
ISG15	-0.76537944	3.25E-05

Table S10. List of significantly upregulated transcripts upon shMEX3A

Genesymbol	logFC	P-value
ANO2	2.229653	3.76E-07
BMP6	1.685296	2.60E-07
OLR1	1.511051	6.32E-07
EPGN	1.459658	3.88E-06
CTH	1.330013	4.35E-07
CPM	1.287348	0.002111
LAMA1	1.259374	5.32E-06
SLC2A3	1.235431	1.35E-06
IL6	1.174748	3.80E-05
TBX3	1.17186	7.50E-07
SLC16A6	1.165492	4.70E-07
BMP2	1.110196	1.11E-06
RIMKLB	1.025333	3.51E-06
SPINK13	1.020487	2.52E-06
DNER	1.013701	7.36E-05
NR4A3	1.0082	1.21E-05
MAP2K6	0.996544	2.78E-05
PHLDB2	0.96662	4.53E-06
NCCRP1	0.963478	5.01E-06
FLVCR2	0.961614	0.00019
PRR5L	0.950251	2.40E-06
SLC2A14	0.950062	1.91E-06
RNF182	0.945418	1.06E-05
FAM134B	0.895902	3.04E-05
NR4A2	0.875728	2.94E-06
